# Inhibition of PPARγ by BZ26, a GW9662 derivate, attenuated obesity-related breast cancer progression by inhibiting the reprogramming of mature adipocytes into to cancer associate adipocyte-like cells

**DOI:** 10.3389/fphar.2023.1205030

**Published:** 2023-08-15

**Authors:** Liangge Li, Jiafeng Geng, Wen Yu, Feifei Zhou, Zhihuan Zheng, Kaiyue Fu, Junjie Kong, Xiujing Feng

**Affiliations:** ^1^ Department of Endocrinology, Key Laboratory of Endocrine Glucose and Lipids Metabolism and Brain Aging, Ministry of Education, Shandong Provincial Hospital Affiliated to Shandong First Medical University, Jinan, Shandong, China; ^2^ School of Clinical and Basic Medical Sciences, Shandong First Medical University and Shandong Academy of Medical Sciences, Jinan, Shandong, China; ^3^ State Key Laboratory of Pharmaceutical Biotechnology, School of Life Sciences, Nanjing University, Nanjing, China

**Keywords:** obesity-related cancer, cancer-associated adipocytes, mature adipocytes, PPARγ modulator, reprogramming

## Abstract

Obesity has been associated with the development of 13 different types of cancers, including breast cancer. Evidence has indicated that cancer-associated adipocytes promote the proliferation, invasion, and metastasis of cancer. However, the mechanisms that link CAAs to the progression of obesity-related cancer are still unknown. Here, we found the mature adipocytes in the visceral fat of HFD-fed mice have a CAAs phenotype but the stromal vascular fraction of the visceral fat has not. Importantly, we found the derivate of the potent PPARγ antagonist GW9662, BZ26 inhibited the reprogramming of mature adipocytes in the visceral fat of HFD-fed mice into CAA-like cells and inhibited the proliferation and invasion of obesity-related breast cancer. Further study found that it mediated the browning of visceral, subcutaneous and perirenal fat and attenuated inflammation of adipose tissue and metabolic disorders. For the mechanism, we found that BZ26 bound and inhibited PPARγ by acting as a new modulator. Therefore, BZ26 serves as a novel modulator of PPARγ activity, that is, capable of inhibiting obesity-related breast cancer progression by inhibiting of CAA-like cell formation, suggesting that inhibiting the reprogramming of mature adipocytes into CAAs or CAA-like cells may be a potential therapeutic strategy for obesity-related cancer treatment.

## 1 Introduction

Obesity is a growing public health concern that affects a significant portion of the global population, and it has been associated with an increased risk of developing a variety of different cancers, such as breast, colon, liver, and prostate cancer (Dirat et al., 2011). Emerging evidence has shown that obesity promoted cancer cell proliferation and metastasis (Ringel et al., 2020; Rathmell, 2021). Importantly, obesity represents a poor predictor of clinical outcomes (Brown, 2021). However, the underlying mechanisms linking obesity to cancer remain elusive. Obesity is associated with high adipose mass and adipose tissue (AT) expansion and dysfunction. AT has been recognized over the years as a significant metabolic and endocrine organ because it secretes hormones, including leptin, adiponectin, and chemokines. Generally, AT is divided into white adipose tissue (WAT) and brown adipose tissue (BAT). WAT is distributed around organ and under skin separately and mainly includes the visceral and subcutaneous WAT, while BAT exists only in the neonatal and early childhood periods (Park et al., 2014a).

Because of their different anatomical positions in the body, WAT and BAT have different characteristics. WAT is best-known for storing excess energy in the form of triglycerides, whereas BAT dissipates chemical energy in the form of heat (Seale et al., 2011). In addition, AT comprises a heterogeneous cell population, including adipocytes, stem cells, macrophages, and other immune cells. Adipocytes are primary stromal cells that are considered to play an active role in the tumor microenvironment. The crosstalk between adipocytes and cancer cells can cause phenotypical and functional changes to both cell types that can further enhance tumor progression. Recent studies demonstrated that the crosstalk between hypoxic adipocytes and stromal vascular cells contributed to tumor development and progression in obese animals (Wu et al., 2019). In Dirat et al. (2010) determined that these adipocytes, known as cancer-associated adipocytes (CAAs), positively contributed to tumor progression, especially in obese women, which explains, at least in part, the poor prognosis observed in this subset of patients. CAAs are found adjacent to cancer cells and communicate with cancer cells by releasing inflammatory factors that can mediate local and systemic effects. These cells have been shown to enter the tumor microenvironment to promote the proliferation, invasion, and metastasis of cancer cells (Rybinska et al., 2021), thereby directly contributing to tumor development and progression (Park et al., 2014b).

Cancer cells reprogram mature adipocytes into CAAs through delipidation and acquiring a fibroblast-like phenotype, which is accompanied by the loss of expression of adipocyte terminal differentiation markers, such as adiponectin, leptin, and fatty acid binding protein (FABP2) and an increase in the secretion of pro-inflammatory cytokines, such as Interleukin 6 (IL-6) and plasminogen activator inhibitor-1 (PAI-1), as well as proteases, such as matrix metalloproteinase 9 (MMP9) and MMP11 (Andarawewa et al., 2005; Dirat et al., 2011). Cancer cells also secrete paracrine signals that can induce lipolysis in adipocytes, causing them to release free fatty acids (FFAs) (Dirat et al., 2011). Interestingly, during the development of obesity, pre-adipocytes differentiate incorrectly, and leptin levels increase, while adiponectin levels decrease (Picon-Ruiz et al., 2017), which is consistent with what occurs within CAAs to enable them to drive cancer progression. For example, one study found that the proliferation of MCF-7 cells increased when the cells were stimulated with the supernatant from mature adipocytes obtained from obese women compared to media that was conditioned with adipocytes from normal or underweight women (Bougaret et al., 2017). In addition, the breast tumor that was induced by the injection of E0771 in the mammary fat pad of mice fed a high-fat diet (HFD) had a larger volume than mice that were fed a normal diet (ND) (Fujisaki et al., 2015). However, questions regarding the roles of CAAs in the development of obesity-related cancer and whether CAAs-like cells exist in obese individuals still need to be answered.

Peroxisome proliferator-activated receptor gamma (PPARγ) is a member of the PPAR family of ligand-inducible transcription factors that have emerged as an attractive pharmacological target for drugs to treat various metabolic disorders such as insulin resistance (Xu et al., 2003), type II diabetes (Saltiel and Olefsky, 1996), and chronic inflammation (Buckingham, 2005). It has been well-documented that PPARγ plays a central role in adipogenesis and obesity-related complications. In particular, the receptor is involved in the occurrence and progression of cancer, and agonist ligands that modulate the activity of PPARγ have been regarded as potential drugs for chemoprevention and the treatment of cancer (Peters et al., 2012). BZ26, a novel derivate of the potent PPARγ antagonist GW9662, was previously considered as a PPARγ modulator by using molecular docking simulations (Bei et al., 2016). In this study, we employed BZ26 to elucidate the molecular mechanisms that link obesity to cancer by understanding how modulating PPARγ activity affects the differentiation of mature adipocytes to CAAs.

## 2 Materials and methods

### 2.1 Reagents

BZ26 was synthesized using a previously published method (Bei et al., 2016). A stock solution (100 mM) of the drug was prepared in DMSO. Lipofectamine™2000 was purchased from Invitrogen (Carlsbad, CA). The PPRE-Luc plasmid and dual-Luciferase reporter assay system were purchased from Promega (Madison, WI, United States). TNF-α, IL-6, CCL2, and IL-1β ELISA kits were purchased from eBioscience (San Diego, CA). Alanine/aspartate aminotransferase (ALT/AST) assay kits, as well as TC, TG, FFA, glucose, and insulin kits, were obtained from Jiancheng Bioengineering Institute (Nanjing, Jiangsu, China). The PAI-1 ELISA kit was purchased from the R&D Systems (United States). DMEM and RPMI1640 media were purchased from Gibco Cell Culture (Thermo Fisher Scientific, Grand Island, NY). All other chemicals were purchased from Sigma-Aldrich (St. Louis, MO), unless otherwise stated.

### 2.2 Mice and treatment

Male C57BL/6J mice (3 weeks old) were purchased from the Animal Genetics Research Center of Nanjing University (Nanjing, China) and housed in a specific-pathogen-free (SPF) facility. The 4 weeks year old mice were fed either a ND consisting of 4.5% fat or an HFD (D12492, 60% fat, 20% carbohydrate, 20% protein, total 5.24 kcal/g; Research Diets Inc., New Brunswick, NJ) for 16 weeks. Then these 20-week year old HFD mice (HFD mice) were grouped (*n* = 9 per group) into five groups to do the dosage dependent experiment and injected with BZ26 (1 mg/kg, 2 mg/kg, or 4 mg/kg), 2 mg/kg GW9662 (Sigma), or saline containing 0.1% DMSO intraperitoneally daily for 3 weeks separately. And for the time course experiment, 2 mg/kg BZ26 were injected into the HFD mice for 1, 2, and 3 weeks, separately. The mice were weighed daily until sacrificed under anesthesia using diethyl ether. Animal welfare and experimental procedures were followed in accordance with the Guide for Care and Use of Laboratory Animals (National Institutes of Health, United States) and the related ethical regulations of Shandong First Medical University.

### 2.3 Mice tumor model

A total of 1 × 10^6^ C57BL/6-derived breast cancer cells from the E0771 murine mammary cancer cell line in Matrigel (Corning, Cat No. 354230) were implanted either in the flank alone or in combination with 2.5 ×10^6^ vehicle- or BZ26-treated mature adipocytes of 20-week year old HFD female mice (*n* = 10 per group). After injection for 7 days, the volumes of the resulting tumors were recorded every 2 days. Finally, after injection 21 days, the tumors were resected and measured.

### 2.4 Mitotic image count

Each specimen was photographed in a 400-fold field of view with a large number of mitotic images. The number of obvious mitotic images on each photograph was counted, and the average value was calculated.

### 2.5 Score of tumor metastasis in lung tissue

Scoring criteria: 0 point, no tumor cell infiltration; 1-point, small tumor metastasis is visible; 2 points; 1-2 flaky tumor metastases can be seen; 3 points, multiple lamellar tumor metastases can be seen.

### 2.6 Isolation of mature adipocytes and SVF

Epididymis (visceral) fat and subcutaneous fat were excised from the mice. One section of the excised fat was frozen at −80°C for extracting RNA, while the other part was minced in Hanks’ Balanced Salt Solution (HBSS; Invitrogen) containing calcium, magnesium, and 0.5% bovine serum albumin (BSA). Collagenase (Type II; Sigma-Aldrich, St Louis, MO) was added to the excited tissue at a final concentration of 1 mg/mL, and the tissue suspensions were incubated at 37°C for 20–30 min with constant shaking. The resulting cell suspensions were filtered through a 100-µm filter and centrifuged at 500 *g* for 10 min to separate the floating adipocytes from the SVF-containing pellet.

### 2.7 Hematoxylin and eosin (H&E) staining

After the mice were sacrificed, the livers, skeletal muscle tissue of legs, and adipose tissues were removed, fixed in phosphate-buffered 10% formalin, and embedded in paraffin blocks. A section from each paraffin block was stained with hematoxylin and eosin (H&E) to examine the pathology of the tissues and to score the infiltration of inflammatory cells for 5–8 sections in a field of view at ×400 magnification (5–6 fields of view per gland per mouse). Scores were given based on the grade of lesion: slight (0.5), mild (1), moderate (2), severe (3), profoundly severe (4), and normal (0) (*n* = 6). Images were acquired using a fluorescence microscopy.

### 2.8 Alanine transaminase aminotransferase (ALT) and aspartate transaminase (AST) activity assay

ALT and AST levels are generally associated with hepatic steatosis and/or inflammation (Renehan et al., 2006). The ALT/AST levels in the blood serum of C57BL/6J mice were assayed using commercially available kits. The absorbance at 510 nm was measured using a Model 680 microplate reader (Bio-Rad Laboratories, Hercules, CA, United States).

### 2.9 Isothermal titration calorimetry (ITC)

ITC experiments were performed at 25°C using a MicroCal ITC200 microcalorimeter (MicroCal Inc., Northampton, MA, United States) as previously described (Feng et al., 2016). The PPARγ protein (5 mg/mL stock in PBS) and the ligand BZ26 (185 Mm stock in DMSO) used in this experiment were diluted by the same dilution buffer. Here, BZ26 stock solutions (185 mM in DMSO) were diluted to 92.5 μM by PBS. PPARγ protein solution (9.25 μM) was dissolved in phosphate buffered saline (PBS, pH = 7.4) containing the DMSO (below 0.05%). The protein was added to the cell, and the ligand solution (10 times more concentrated than the protein solution) was injected into the cell in 19 aliquots of 20 μL each for 4 s (the first injection was 0.4 μL for 0.8 s) with delay intervals of 180 s between injections. A reference titration of only the ligand in buffer was used to correct for the heat of dilution. The syringe stirring speed was set at 1,000 rpm. The thermodynamic data were processed with Origin 7.0 software provided by MicroCal. To correct for any discrepancies in the baseline outlined by the software, a manual adjustment was performed.

### 2.10 Quantitative real-time PCR

Total RNA was extracted from macrophages and reverse-transcribed into cDNA using the BioTeke Supermo III RT Kit (BioTeke Corporation, Beijing, China). Quantitative RT-PCR was employed to measure the changes in the mRNA expression levels of mouse Fabp2, leptin, adiponectin, Mmp-9, Mmp-11, and Pai-1; lipid droplet formation-related genes *Cidea*, *Plin2*, *Fitm1*, *Fitm2*, and *G0s2*; lipid uptake-related genes *Fabp1* and *Lpl*; fatty oxidation genes *Mcpt1*, *Pdk4*, *Acox1*, and *Acaa2*; lipogenesis-related genes *Fasn*, *Scd1*, *Hmgcr*, *Acaca*, and *Nrob2*; brown adipose identity genes *Pparα*, *Otop1*, and *Cidea*; thermogenic genes *Pgc1-α*, *Dio2*, and *Ucp1*; and mitochondrial electron transport genes *Cox3*, *Cox5b,* and *Cox8b* using a iCycler thermocycler system and iQ5 optical system (Bio-Rad). Threshold cycle numbers were determined using the iCycler thermocycler system software version 1.0. PCR cycling conditions comprised 1 cycle at 94°C for 5 min, followed by 40 cycles at 94°C for 30 s, 60°C for 30 s, and 72°C for 45 s. The primers used are listed in Table 1. Relative mRNA expression levels of target genes was calculated by normalizing to the control group and the level of β-actin using the 2^−ΔΔCT^ method (Livak and Schmittgen, 2001).

**TABLE 1:** Sequences of the primers used for qRT-PCR.

Gene	Forward primer	Reverse primer
IL-1b	CTT​CAG​GCA​GGC​AGT​ATC​ACT​C	TGC​AGT​TGT​CTA​ATG​GGA​ACG​T
IL-6	ACA​ACC​ACG​GCC​TTC​CCT​AC	TCT​CAT​TTC​CAC​GAT​TTC​CCA​G
Tnf-a	CGA​GTG​ACA​AGC​CTG​TAG​CCC	GTC​TTT​GAG​ATC​CAT​GCC​GTT​G
β-Actin	TGC​TGT​CCC​TGT​ATG​CCT​CT	TTT​GAT​GTC​ACG​CAC​GAT​TT
Fizz1	AGG​AGC​TGT​CAT​TAG​GGA​CAT​C	GGA​TGC​CAA​CTT​TGA​ATA​GG
Ym1	AGA​AGG​GAG​TTT​CAA​ACC​TGG​T	GTC​TTG​CTC​ATG​TGT​GTA​AGT​GA
Arg-1	CTC​CAA​GCC​AAA​GTC​CTT​AGA​G	AGG​AGC​TGT​CAT​TAG​GGA​CAT​C
CCR2	ATG​CAA​GTT​CAG​CTG​CCT​GC	ATG​CCG​TGG​ATG​AAC​TGA​GG
NOS2	GCT​TCT​GGT​CGA​TGT​CAT​GAG	TCC​ACC​AGG​AGA​TGT​TGA​AC
Adiponectin	GGA​ACT​TGT​GCA​GGT​TGG​AT	GCT​TCT​CCA​GGC​TCT​CCT​T
Cidea	ATC​ACA​ACT​GGC​CTG​GTT​ACG	TAC​TAC​CCG​GTG​TCC​ATT​TCT
Cox8b	GAA​CCA​TGA​AGC​CAA​CGA​CT	GCG​AAG​TTC​ACA​GTG​GTT​CC
Cox3	CAA​GGC​CAC​CAC​ACT​CCT​ATT	GTC​AGC​AGC​CTC​CTA​GAT​CA
Cox5b	TCT​AGT​CCC​GTC​CAT​CAG​CA	AGA​CAT​TCT​GTG​AGG​CAG​GT
Pgc-1a	CCC​TGC​CAT​TGT​TAA​GAC​C	TGC​TGC​TGT​TCC​TGT​TTT​C
Ucp1	CAC​CTT​CCC​GCT​GGA​CAC​T	CCC​TAG​GAC​ACC​TTT​ATA​CCT​AAT​GG
Fabp4	GCT​TTT​GTA​GGT​ACC​TGG​AAA​CTT	ACA​CTG​ATG​ATC​ATG​TTA​GGT​TTG​G
*Dio2*	TAC​AAA​CAG​GTT​AAA​CTG​GGT​GAA​GAT​GCT​C	GAG​CCT​CAT​CAA​TGT​ATA​CCA​ACA​GGA​AGT​C
PPARα	AAC​ATC​GAG​TGT​CGA​ATA​TGT​GG	CCG​AAT​AGT​TCG​CCG​AAA​GAA
Otop1	ACT​CTC​TGG​TTG​ACA​GTC​GC	TGT​GAG​TCT​CCA​CTT​GCA​CC
Leptin	GTG​GCT​TTG​GTC​CTA​TCT​GTC	CGT​GTG​TGA​AAT​GTC​ATT​GAT​CC
MMP-9	GGA​CCC​GAA​GCG​GAC​ATT​G	CGT​CGT​CGA​AAT​GGG​CAT​CT
MMP-11	CCA​CTC​ACT​TTC​ACT​GAG​GTG	CGT​CAA​ACG​GCA​AGT​TGT​CAC
FABP2	GTG​GAA​AGT​AGA​CCG​GAA​CGA	CCA​TCC​TGT​GTG​ATT​GTC​AGT​T
PAI-1	CAA​GCT​CTT​CCA​GAC​TAT​GGT​G	ACC​TTT​GGT​ATG​CCT​TTC​CAC

### 2.11 PPARγ gene-reporter luciferase assay

HEK293 cells were transfected with either pIRES-hPPARγ, or mutated vectors and PPRE-Luc or pRL-control using the Lipofectamine 2000 transfection reagent. The mutated vectors (i.e., pcDNA3.1 (−)-PPARγ2 (aa1-505), pcDNA3.1 (−)-PPARγ2-DBD-AF2 (aa137-505), pcDNA3.1 (−) -PPARγ2-hinge-AF2 (aa211-505), and pcDNA3.1 (−)-PPARγ2-LBD (aa319-505) vectors were generated from the Plenti-hPPARγ plasmid as previously reported (Feng et al., 2016). The plasmid pRL containing *Renilla* luciferase cDNA was used as an internal control reporter. After 24 h transfection, luciferase activities were measured using the Dual-Luciferase reporter assay system. The *Renilla* luciferase activity was normalized to the firefly luciferase activity.

### 2.12 Fat transcriptome analysis by RNA-seq

Three samples of subcutaneous and visceral fat from ND-fed and 20-week year old HFD-fed mice were sent to Novogene (Beijing, China) for RNA sequencing. Firstly, RNA degradation and contamination were monitored on 1% agarose gels. Then RNA purity was checked using the NanoPhotometer^®^ spectrophotometer (IMPLEN.CA.United States), and RNA integrity was assessed using the RNA Nano 6000 Assay Kit of the Bioanalyzer 2100 system (Agilent Technologies, CA, United States). For the data analysis, clean data were provided by the company; paired-end reads were mapped to the mouse genome (GRCm38 (mm10)) and assembled by TopHat Aligment and Cufflinks Pipeline from Illumina BaseSpace as a default setup. Differential expression analysis of two groups was performed using the DESeq2 R package (1.20.0). DESeq2 provide statistical routines for determining differential expression in digital gene expression data using a model based on the negative binomial distribution. Genes with an adjusted *p*-value<0.05 found by DESeq2 were assigned as differentially expressed. Gene Ontology (GO) enrichment analysis of differentially expressed genes was implemented by the clusterProfiler R package, in which gene length bias was corrected. GO terms with corrected *p*-value less than 0.05 were considered significantly enriched by differential expressed genes. KEGG is a database resource for understanding high-level functions and utilities of the biological system from the molecular-level information. We used clusterProfiler R package to test the statistical enrichment of differential expression genes in KEGG pathways.

### 2.13 Data analyses and statistics

The data reported herein are expressed as the mean ± standard error of mean (SEM). The statistical analysis was performed by Student’s t-test when only two value sets were compared. A one-way analysis of variance (ANOVA) followed by a Tukey’s multiple comparisons test were used when the data involved three or more groups. *p < 0.05, **p < 0.01, or ***p < 0.001 were considered statistically significant.

## 3 Results

### 3.1 CAA-associated genes were highly expressed in the visceral fat of HFD-fed mice

**FIGURE 1:**
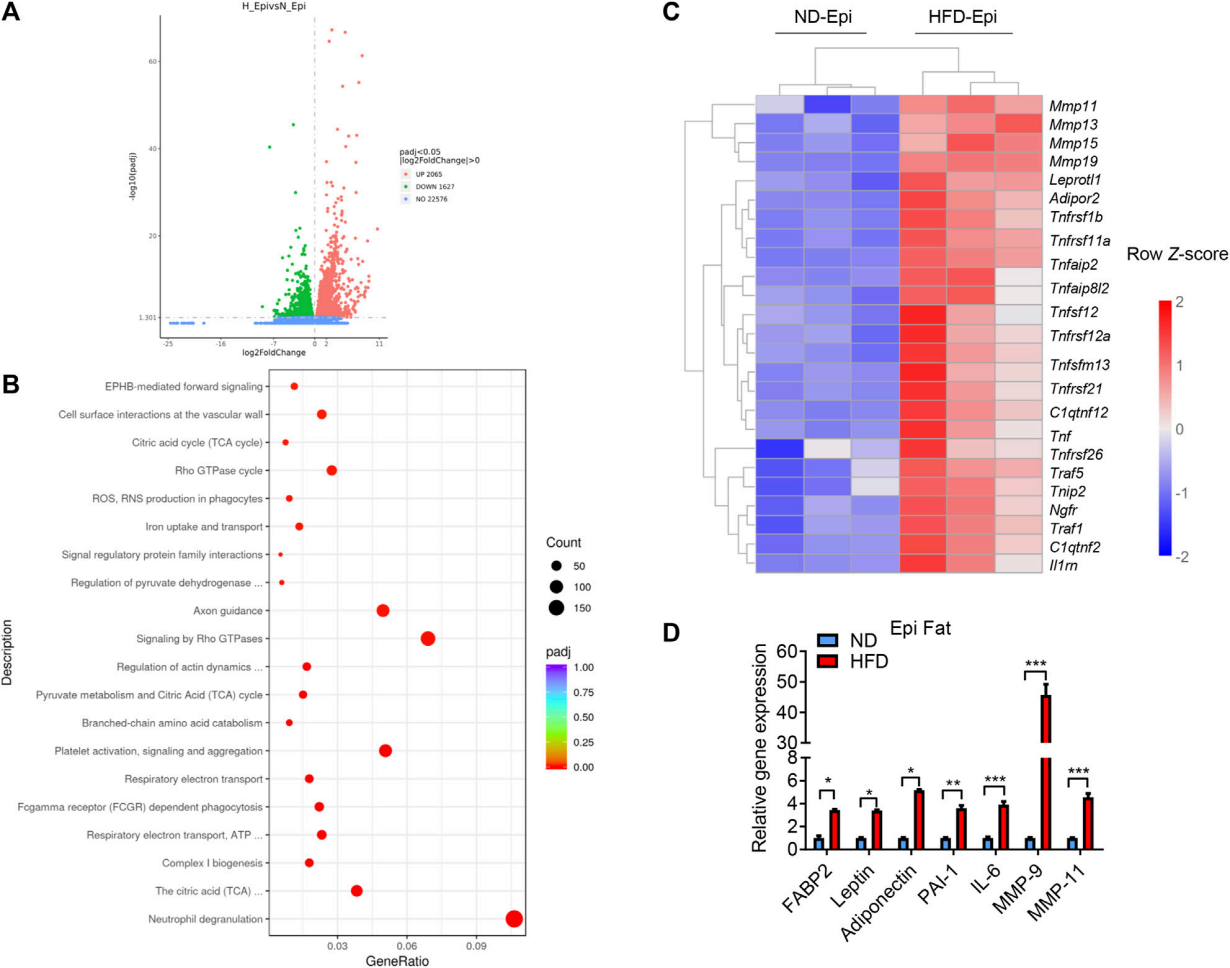
CAA associated genes were highly expressed in the visceral fat of HFD-fed mice. **(A)** Volcano plot of the up- and downregulated genes in the visceral fat of HFD-fed mice highlighted in red and green, respectively. **(B)** Dot plot of significantly enriched Reactome pathways in the visceral fat of HFD-fed and ND-fed mice. Log2FC distributions of member genes are plotted on the *x*-axis. The color of the distributions represents the *p*-value of the enriched pathway. **(C)** Heatmap depicting the levels of the differentially expressed CAAs-related genes using RNA-Seq from the visceral fat of ND-fed and HFD-fed mice. **(D)** Levels of CAA-related genes in the visceral fat of ND-fed and HFD-fed mice quantified by qRT-PCR.

Obesity is a principal risk factor of cancer, and several different cancers are known to occur in areas rich in adipose tissue. CAAs that lie adjacent to cancer cells in the tumor microenvironment are thought to be involved in the development and metastasis of cancer. We were interested in elucidating whether CAAs or CAAs-like cells are present in adipose tissue of obese mice to establish a clearer link between obesity and cancer. Firstly, we isolated the visceral (epididymis) fat from ND-fed and HFD-fed mice and subjected the AT to RNA-Seq. The results showed that, in the visceral fat, there were 3692 differentially expressed genes (DEGs) between the mice in the ND and HFD groups, of which 2065 genes were upregulated and 1627 genes were downregulated (Figure 1A). Inputting the bioinformatics data into the Kyoto Encyclopedia of Genes and Genomes (KEGG) database, the genes associated with classification of biological pathways of different genes between the ND and HFD group were enriched. The enriched pathways were divided into 20 categories, including neutrophil degranulation, signaling by Rho GTPase, axon guidance, platelet activation, signaling and aggregation, cell surface interactions at the vascular wall, citric acid cycle (TCA-cycle), Rho GTPase cycle, ROS, reactive oxygen and nitrogen species production in phagocytes, iron uptake and transport, signal regulatory protein family interactions, EPHB-mediated forwards signaling, focal adhesion, Rap1 signaling pathway, regulation of pyruvate dehydrogenase, complex I biogenesis, the citric acid (TCA) and respiratory electron transport, ATP (Figure 1B). And in order to evaluate the expression difference of CAA related genes in the AT of ND and HFD mice, a heat map was generated to visualize the significant expression differences between the AT of ND and HFD groups (Figure 1C). Meanwhile, the RT-qPCR was used to verify the sequencing result and showed that CAA-related genes were more highly expressed in the visceral fat of HFD mice compared to the ND mice (Figure 1D), which suggested that CAAs or CAA-like cells are indeed present in the epididymal fat of HFD mice.

### 3.2 BZ26 inhibited the formation of CAA-like cells in the HFD-fed mice

**FIGURE 2:**
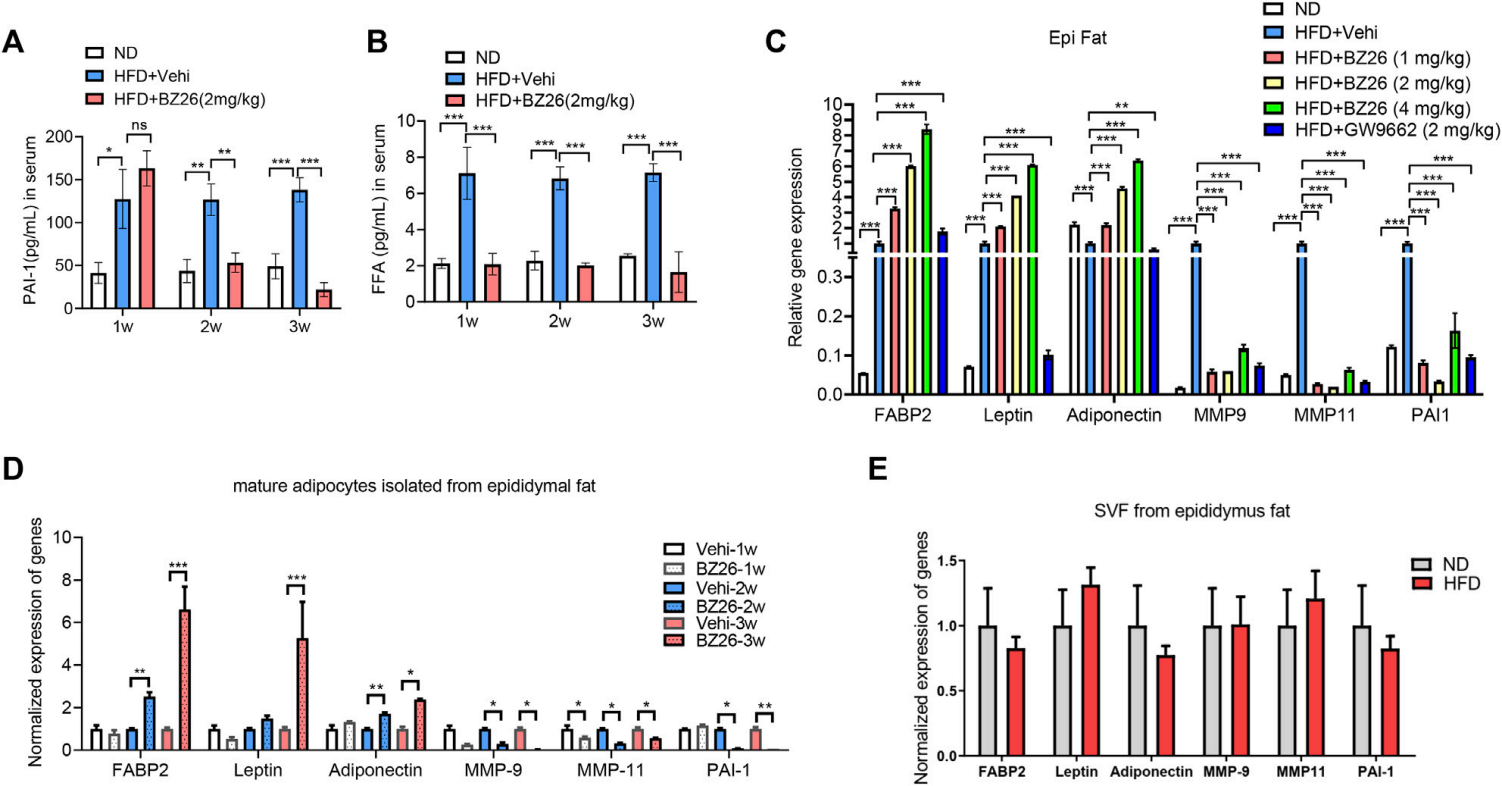
BZ26 inhibited the reprogramming of mature adipocytes into CAA-like cells in the visceral fat of HFD-fed mice. **(A)** FFA levels in the serum of the HFD-fed mice and the 2 mg/kg BZ26 treatment group. **(B)** PAI-1 levels in the serum of the HFD-fed mice and the 2 mg/kg BZ26 treatment group. **(C)** Expression levels of CAA marker genes encoding FABP2, leptin, adiponectin, IL-6, PAI-1, MMP-9, and MMP11 in the visceral fat tissue of HFD-fed mice and BZ26-treated HFD-fed mice assayed by qRT-PCR. **(D)** Expression levels of CAA marker genes encoding FABP2, leptin, adiponectin, IL-6, PAI-1, MMP-9, and MMP11 in the mature adipocytes of HFD-fed mice and BZ26-treated HFD-fed mice assayed by qRT-PCR. **(E)** Expression levels of CAA marker genes encoding FABP2, Leptin, Adiponetin, MMP-9, MMP-11 and PAI-1 in the SVF from the epididymus fat of HFD and ND mice assayed by qRT-PCR.

Recently, significant attention has been devoted to optimizing the structure-activity relationships of PPARγ ligands to decrease or abrogate their side effects. Modification of existing structures to increase potency and selectivity also represents a promising strategy for developing new, safer ligands or modulators of PPARγ (Doshi et al., 2010; Feng et al., 2016). During the screening of small molecules that regulate obesity-related metabolic syndrome, BZ26, a novel GW9662 derivate that was identified as a potential ligand of PPARγ via molecular docking studies (Bei et al., 2016). To assess whether BZ26 affected the formation of CAA-like cells *in vivo*, male C57BL/6J mice were fed a HFD for 16 weeks, during which the mice gradually became obese. Then, the mice were treated daily with either 1 mg/kg, 2 mg/kg, or 3 mg/kg BZ26 or vehicle for 3 weeks, GW9662, a PPARγ antagonist, was used as a control. For the time course experiment, 2 mg/kg BZ26 treated the HFD mice for 1 week, 2 or 3 weeks, separately. After BZ26 was administered to the HFD-fed mice, the epididymis fat and the serum of the mice was analyzed for CAA phenotype-related genes and the other CAA markers. The level of PAI-1 in the serum and AT of HFD mice was higher than the ND group. However, the higher protein level of PAI-1 in the serum were inhibited by 2 mg/kg BZ26 treatment for 2 or 3 weeks (Figure 2A). And its mRNA expression in the AT of HFD group was significantly inhibited by different dosage of BZ26 treatment for 3 weeks (Figure 2C). Consistently, the levels of FFA in the serum and visceral fat were also lowered by BZ26 (Figure 2B). In addition, the expressions of CAA markers, including adipocyte terminal differentiation markers (FABP2, leptin, adiponectin), inflammatory cytokine PAI-1, and proteases (MMP-9, MMP11) were upregulated in the AT of the HFD-fed mice that were treated with BZ26 (Figure 2C).

AT comprises a heterogeneous cell population, mostly comprising adipocytes but also various other stromal cells, including endothelial cells, pericytes, macrophages, and adipocyte progenitor cells. Both adipocytes and the stromal vascular cells in the AT contribute to tumor development and progression (Iyengar et al., 2003). Therefore, we isolated the mature adipocytes in the visceral fat from the obese mice and analyzed their phenotypes by measuring the expression levels of CCA markers by qRT-PCR. The data shown in Figure 2D indicated that the expression levels of CCA markers in the mature adipocytes were lower in the HFD-fed mice treated with BZ26 in a time-dependent manner compared to the abnormally high levels of these markers in the HFD-fed mice. Importantly, to test whether the preadipocytes had the same phenotype as the mature adipocytes, we isolated the SVF from the visceral fat and measured the expression levels of CAA marker genes by qRT-PCR. The expression of FABP2, Leptin, Adiponectin, MMP-9, MMP-11, and PAI-1 has no significant change between the SVF of HFD-fed mice and ND-fed mice, suggesting that the SVF did not have the CAA phenotype (Figure 2E). Taken together, these results indicated that BZ26 promoted the reprogramming of mature adipocytes in the visceral fat of the HFD-fed mice to a CAA-like phenotype, but this did not occur with the preadipocytes.

### 3.3 BZ26 inhibited obesity-related breast cancer progression

**FIGURE 3:**
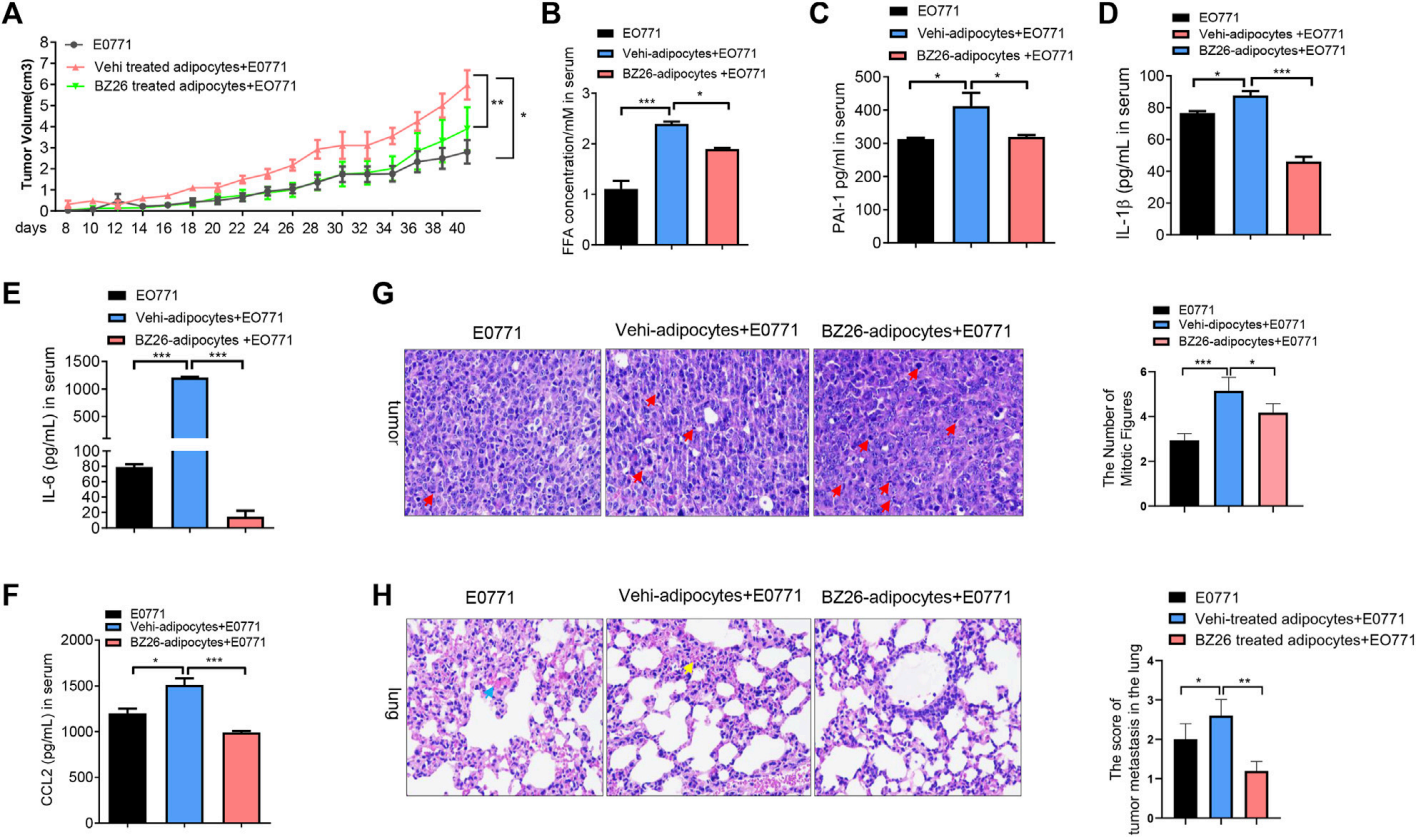
BZ26-treated mature adipocytes inhibited breast cancer proliferation and metastasis in HFD-fed mice. **(A)** The volume of the tumor in HFD mice injected E0771 only or (E0771+adipocyte mixture) or (BZ26-treated adipocytes + E0771) mixture. **(B–F)** Levels of FFAs,PAI-1, IL-1β, IL-6 and CCL-2 in the serum of HFD mice injected E0771 only or (E0771 + adipocyte mixture) or (BZ26-treated adipocytes + E0771) mixture. **(G)** H&E staining and quantification of the numbers of mitotic figures (×200 magnification) of the tumor tissue isolated from the HFD mice injected E0771 only or (E0771 + adipocyte mixture) or (BZ26-treated adipocytes + E0771) mixture. **(H)** H&E staining of tumor tissue and quantification of the tumor metastasis in the lung tissue. Scoring criteria: 0, no tumor cell infiltration; 1, small tumor metastasis; 2, 1–2 flaky tumor metastases; 3, multiple flaky tumor metastases (×200 magnification).

Cancer cells cooperate with other adjacent cells to promote tumor growth and invasion. Given that BZ26 had the ability to inhibit the differentiation of mature adipocytes in the visceral fat of obese mice to a CAA-like phenotype, we sought to determine whether BZ26 could inhibit the development of obesity-related cancer by inhibiting the formation of CAAs. To accomplish this, we isolated mature adipocytes from HFD-fed mice and BZ26-treated HFD-fed mice, mixed the adipocytes with breast cancer cells (E0771 cell line), and then injected the mixture into the mammary tissue of the mice. The tumor sizes in the mice in the mixture group (i.e., E0771 cancer cells and mature adipocytes) were larger than the tumor sizes in the mice which only the E0771 cells were injected (Figure 3A). As expected, BZ26 treatment led to a reduction in the sizes of the tumors compared with the mixture group, suggesting that the mature adipocytes promoted tumor growth and that BZ26 could reverse this effect. Consistently with this, the serum FFA and PAI-1 levels, which were higher in the mice in the mixture group relative to the control mice, were lower in the BZ26-adipocyte group compared with mixture group (Figures 3B, C). In addition, the levels of inflammatory factors, such as IL-1β, IL-6, and CCL2, which were markedly higher in the mixture group compared to the control group, were reduced after BZ26 treatment (Figures 3D–F).

Mitotic figures are manifestations of tumor cell atypia. Benign tumors have little mitotic figures, no pathological mitotic figures, while malignant tumors have more, visible pathological mitotic figures. To evaluate the effect of BZ26 on tumor progression, H&E assays and quantification of the mitotic figures were performed in the tumor tissue of the mice. Compared to the E0771 group, more mitotic figures were observed in the tumor tissue of the mice in the mixture, but BZ26 treatment induced a significant decrease in the number of mitotic figures (Figure 3G), suggesting that mature adipocytes promoted tumor malignancy, while BZ26 attenuated this effect. Moreover, breast cancer is prone to lung metastasis, so we assessed tumor metastasis in the lung tissue by H&E staining and scored the tumor infiltration. Consistently, BZ26 suppressed tumor metastasis into the lung tissue (Figure 3H). Taken together, these results substantiated that mature adipocytes promoted the growth of breast tumors and metastasis, but BZ26 could reduce the pathological burden.

### 3.4 BZ26 mediated the browning of visceral and subcutaneous fat

**FIGURE 4:**
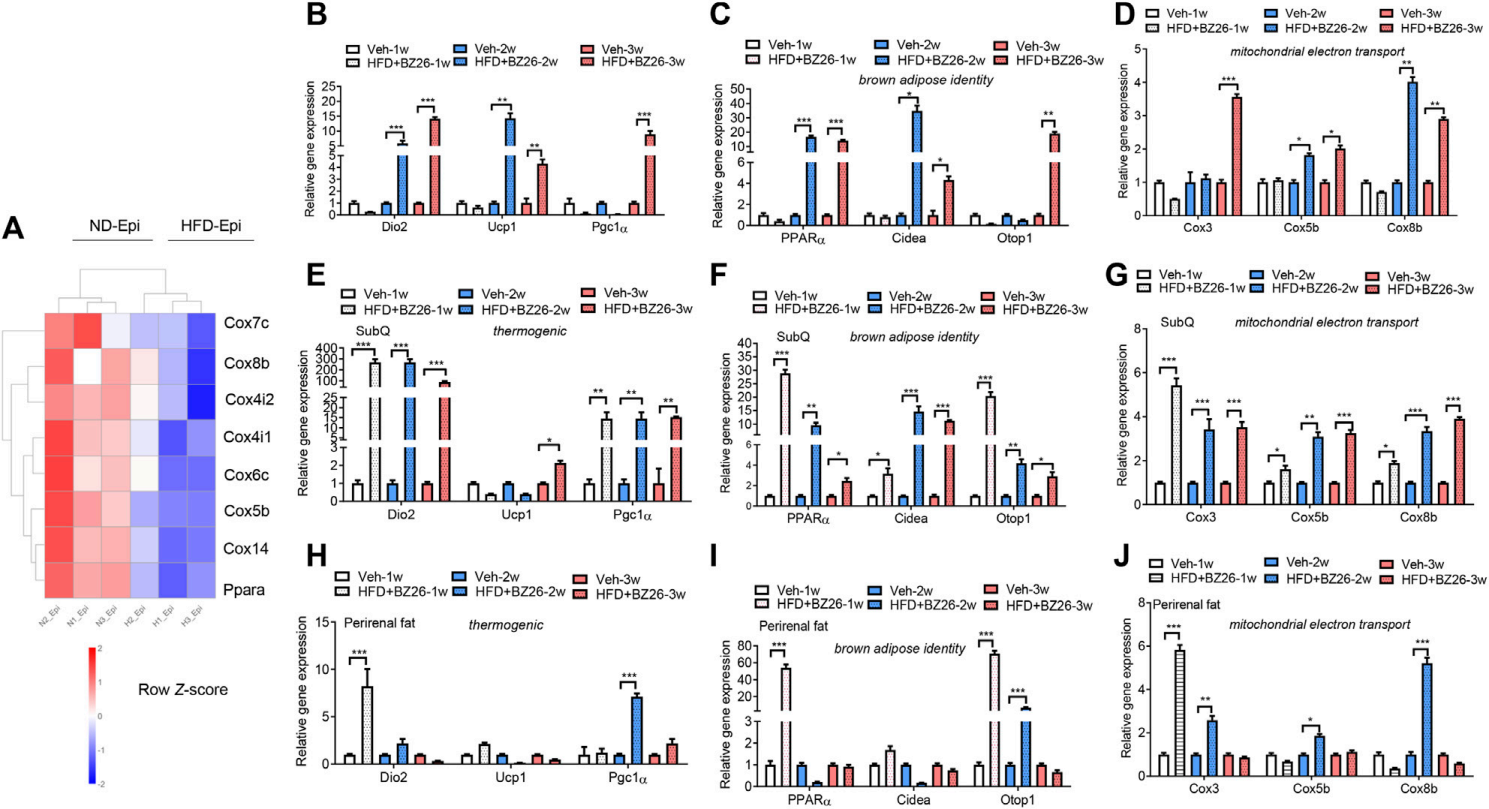
BZ26 mediated the browning of visceral fat. **(A)** Heatmap depicting the levels of differentially expressed genes involved in the browning of adipose tissue. **(B)** mRNA expression levels of thermogenic genes, including *Dio2*, *Ucp1*, and *Pgc1α*, in the visceral fat of HFD-fed mice with and without treatment with 2 mg/kg BZ26 for one, two, and 3 weeks. **(C)** mRNA expression levels of brown adipose-related genes, such as *PPARα*, *Cidea*, and *Otop1*, in the visceral fat of HFD-fed mice with and without treatment with 2 mg/kg BZ26 for one, two, and 3 weeks. **(D)** The mRNA expression levels of mitochondrial electron transport-related genes, such as *Cox3*, *Cox5b*, and *Cox8b*, in the visceral fat of HFD-fed mice with and without treatment with 2 mg/kg BZ26 for one, two, and 3 weeks. **(E)** mRNA expression levels of thermogenic genes, including *Dio2*, *Ucp1*, and *Pgc1α*, in the subcutaneous fat of HFD-fed mice with and without treatment with 2 mg/kg BZ26 for one, two, and 3 weeks. **(F)** mRNA expression levels of brown adipose-related genes, such as *PPARα*, *Cidea*, *Otop1*, in the subcutaneous fat of HFD-fed mice with and without treatment with 2 mg/kg BZ26 for one, two, and 3 weeks. **(G)** mRNA expression levels of mitochondrial electron transport-related genes, such as *Cox3*, *Cox5b*, and *Cox8b*, in the subcutaneous fat of HFD-fed mice with and without treatment with 2 mg/kg BZ26 for one, two, and 3 weeks. **(H)** mRNA expression levels of thermogenic genes, including *Dio2*, *Ucp1*, and *Pgc1α*, in the perirenal fat of HFD-fed mice with and without treatment with 2 mg/kg BZ26 for one, two, and 3 weeks. **(I)** mRNA expression levels of brown adipose-related genes, such as *PPARα*, *Cidea*, *Otop1*, in the perirenal fat of HFD-fed mice with and without treatment with 2 mg/kg BZ26 for one, two, and 3 weeks. **(J)** mRNA expression levels of mitochondrial electron transport-related genes, such as *Cox3*, *Cox5b*, and *Cox8b*, in the perirenal fat of HFD-fed mice with and without treatment with 2 mg/kg BZ26 for one, two, and 3 weeks.

Traditionally, AT has been divided into two types: WAT, best known for storing excess energy in the form of triglycerides, and BAT, which oxidizes chemical energy to produce heat to protect against hypothermia and obesity (Kajimura et al., 2008; Seale et al., 2008; Seale et al., 2011). Here, we assessed how BZ26 affected the phenotype of adipocytes in HFD-fed mice. First, the RNA-Seq data showed that adipose tissue browning-related genes such as Cox7c, Cox8b, Cox4i2, Cox4i2, Cox6c, Cox5b, Cox14, and PPARα were significant decreased in the visceral fat (epididymis fat) of HFD group compared to the ND group (Figure 4A). Then, the epididymis fat was collected from the HFD-fed mice and BZ26-treated HFD-fed mice and analyzed for its browning characteristics. This was accomplished by measuring the changes in mRNA expression levels of key thermogenic genes (*Dio2* (type II iodothyronine deiodinase)*, Pgc1α* (peroxisome proliferator-activated receptor gamma, coactivator 1α)*,* and *Ucp1* (uncoupling protein 1); brown adipose-related genes [*Cidea* (cell death-inducing DNA fragmentation factor, alpha subunit-like effector a)*, Otop1* (otopetrin 1), and *PPARα* (peroxisome proliferator activator receptor α)]; and mitochondrial electron transport-related genes [*Coxiii* (cytochrome c oxidase subunit 3)*, Cox5b* (cytochrome c oxidase subunit 5b), and *Cox8b* (cytochrome c oxidase subunit 8b)], which are classical markers of BAT, in the epididymis fat of the mice in each group were measured by RT-qPCR.

After the HFD-fed mice were treated with 2 mg/kg BZ26 for 1 week, 2 weeks, or 3 weeks, the mRNA levels of *Dio2* and *Pgc1α* were significantly higher compared to the levels in the control group (Figure 4B). However, the mRNA levels of *Ucp1* were lower than in the control group for the first 2 weeks of BZ26 treatment but then increased after being treated with BZ26 for 3 weeks. All thermogenic gene expression in the epididymis AT was lower than HFD group after being treated with BZ26 for 1 week, but expression of these genes was upregulated in the BZ26-treated HFD-fed mice after 3 weeks (Figure 4C). The mRNA expression of the brown adipose-related genes (*PPARα*, *Cidea,* and *Otop1*) was gradually upregulated in the BZ26-treated HFD-fed mice compared to the HFD-fed mice (Figure 4C). Furthermore, the expression levels of the mitochondrial electron transport genes *Coxiii, Cox5b,* and *Cox8b* were significantly increased by BZ26 in the visceral AT (Figure 4D). The subcutaneous fat and perirenal fat (peri fat) were also isolated from the ND-fed and HFD-fed mice to analyze their browning characteristics, including the thermogenic gene (*Ucp1*, *Dio2* and *Pgc1α*), brown adipose-related genes (*PPARα*, *Cidea* and *Otop1*) and the mitochondrial electron transport genes (*Coxiii, Cox5b,* and *Cox8b*). The results suggested that BZ26 also induced a “browning” of the subcutaneous (Figures 4E–G) and peri fat (Figures 4H–J). Taken together, these results indicated that BZ26 induced the browning of visceral and subcutaneous fat.

### 3.5 BZ26 inhibited adipose tissue inflammation in HFD-fed mice

**FIGURE 5:**
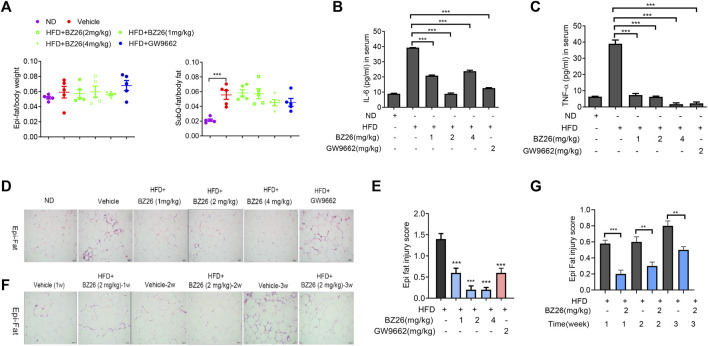
BZ26 induced the immunosuppression of adipose tissue in HFD-fed mice. **(A)** Weights of the visceral and subcutaneous fat. **(B)** Levels of IL-6 in the serum of ND-fed, HFD-fed, BZ26-treated HFD-fed mice separately, measured by ELISAs. **(C)** Levels of TNF-α in the serum of ND-fed, HFD-fed, BZ26-treated HFD-fed mice separately, measured by ELISAs. **(D)** H&E staining to determine the infiltration of inflammatory cells in the visceral fat of HFD-fed mice and HFD-fed mice treated with 1, 2, and 4 mg/kg BZ26 for 3 weeks. (*n* = ×5, ×200 magnification). **(E)** Quantification of inflammatory cell infiltration in the visceral fat. **(F)** H&E staining to determine the infiltration of inflammatory cell in the visceral fat of HFD-fed mice and HFD-fed mice treated with 2 mg/kg BZ26 for one, two, and 3 weeks (n = ×5, ×200 magnification). **(G)** Quantification of inflammatory cell infiltration in the visceral fat.

AT is not only a reservoir for energy but also an immune organ. In the context of obesity, AT inflammation eventually leads to chronic inflammation and metabolic diseases. First, we analyzed the effect of BZ26 on the weight of visceral fat and subcutaneous fat in the HFD-fed mice, and the data shown in Figure 5A indicated that BZ26 had no significant effect on the weights of the visceral and subcutaneous fat. However, BZ26 did inhibit the release of inflammatory cytokines such as IL-6 and TNF-α into the serum (Figures 5B, C). Next, inflammatory cell infiltration and AT injury in the visceral (Epi) fat tissue of the mice were assessed by H&E assay. The visceral fat of the mice that were fed a HFD contained high levels of infiltrated inflammatory cells, but the infiltration was significantly inhibited by BZ26 in a time-independent manner (Figures 5D, E) and dose-independent manner (Figures 5F, G). Taken together, BZ26 ameliorated the HFD-induced inflammation of AT.

### 3.6 Obesity-related metabolic disorders are attenuated by BZ26

**FIGURE 6:**
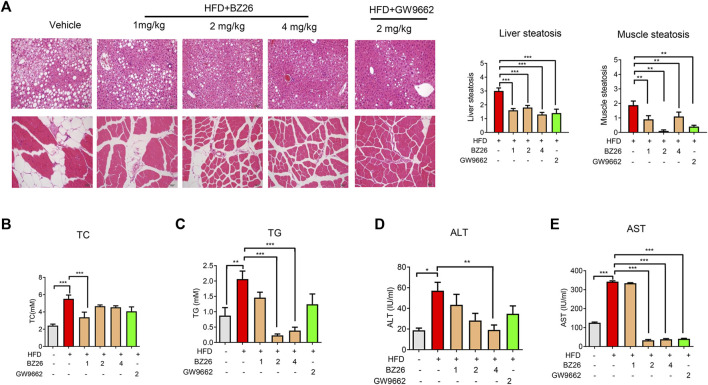
BZ26 attenuated the metabolic disorders. **(A)** H&E staining and quantification of hepatic/muscular steatosis from 5–8 tissue sections/400× field, 5–6 fields/gland/mouse fed a HFD and treated with the vehicle (0.1% DMSO), BZ26, and GW9662 (2 mg/kg) for 3 weeks. The grade of lesion was scored as slight (0.5), mild (1), moderate (2), severe (3), profound severe (4), and normal (0), (*n* = 6). All values are expressed as the mean ± SEM. Statistical analysis was based on one-way ANOVA followed by a Dunnett’s test. **p < 0.01, ***p < 0.01 compared to the vehicle. **(B)** Levels of TC and TG and **(C)** activities of ALT **(D)** and AST **(E)** in the serum collected from HFD-fed mice with and without BZ26 treatment (*n* = 9 or 6). All values are expressed as the mean ± SEM. Statistical analysis was based on one-way ANOVA followed by a Dunnett’s test or Student’s t-test for comparing two groups. ***p < 0.001 compared to the vehicle.

Metabolic dysfunction is a key risk factor for obesity-related cancer. To evaluate the effect of BZ26 on obesity-induced metabolic syndrome, the livers and muscle tissue were isolated from the mice and subjected to histological analysis by H&E staining. As shown in Figure 6A, various degrees of liver and muscular steatosis were observed in all HFD-fed mice. Cells containing many lipid droplets of different sizes were observed throughout the hepatic lobules and muscle tissue. However, the derangement of cell structures and excessive lipid droplets observed in the liver and muscle cells of the HFD-fed mice was significantly alleviated by all BZ26 treatment group for 3 weeks. Moreover, the total triglyceride (TG) level in the serum of HFD-fed mice was significantly reduced after treatment by BZ26, and the high total cholesterol (TC) level in the serum of the HFD-fed mice was reduced after treatment with 1 mg/kg BZ26, but not obvious in the 2 mg/kg BZ26 and 4 mg/kg BZ26 group (Figures 6B, C). In addition, high ALT and AST levels in serum are generally associated with the hepatic steatosis and/or inflammation (Sheth et al., 1997). The ALT and AST levels in the serum of the HFD-fed C57BL/6J mice were reduced by BZ26 treatment (Figures 6D, E). Taken together, these data suggested that BZ26 significantly alleviated HFD-induced liver and muscle steatosis and metabolic disorders.

### 3.7 BZ26 directly binds with PPARγ

**FIGURe 7:**
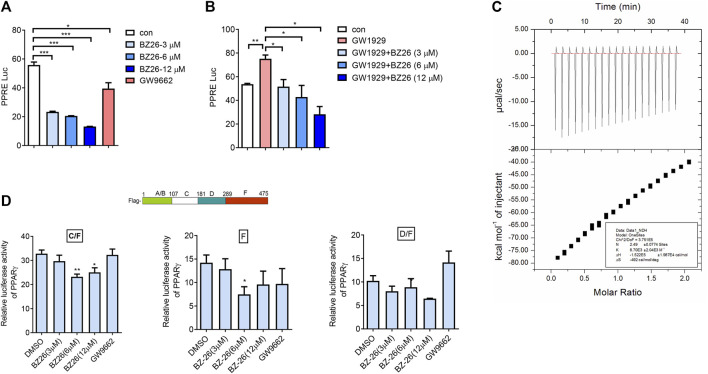
BZ26 is a modulator of PPARγ activity. **(A)** Transcriptional activation of PPARγ in cells treated with the indicated doses of BZ26 or GW9662. HEK293T cells were transfected with pIRES-mPPARγ/PPRE and pRL-control using Lipofectamine 2000. Then, cells were pretreated with apigenin for 24 h. Luciferase activities were measured using the dual-luciferase reporter assay system. All values are expressed as the mean ± SEM. Statistical analysis was based on one-way ANOVA followed by a Dunnett’s test. *p < 0.05, ***p < 0.001 compared to the control group. **(B)** Transcriptional activation of PPARγ in cells treated with the indicated doses of GW1929 or GW1929 plus BZ26. HEK293T cells were transfected with pIRES-mPPARγ/PPRE and pRL-control using Lipofectamine 2000. Then, the cells were pretreated with apigenin for 24 h. Luciferase activities were measured by using the dual-luciferase reporter assay system. All values are expressed as the mean ± SEM. Statistical analysis was based on one-way ANOVA followed by a Dunnett’s test. *p < 0.05, ***p < 0.001 compared to the control group. **(C)** ITC data for binding of BZ26 to PPARγ. The upper panels show the raw data, and the lower panels show the corresponding binding isotherm fitted to a one-binding-site model. A reference titration of the ligand into buffer was used to correct for the heat of dilution. The thermodynamic parameters (K, ΔH, and ΔS) are indicated. **(D)** HEK293T cells were transfected with pIRES-mPPARγ truncated mutants/PPRE and pRL-control using Lipofectamine 2000. Then, cells were pretreated with BZ26 (3 μM, 6 μM, and 12 µM) for 24 h. Luciferase activities were measured using the dual-luciferase reporter assay system. All values are expressed as the mean ± SEM. Statistical analysis was based on one-way ANOVA followed by a Dunnett’s test. *p < 0.05, **p < 0.01 compared to the control group.

Bei et al. (2016) used molecular docking studies and found that BZ26 binds to the ligand-binding domain of PPARγ. Here, the effect of BZ26 on PPARγ activity was evaluated by luciferase reporting assays and the data showed that PPARγ activity was markedly inhibited by BZ26 in a dose-independent manner, and its efficacy in inhibiting PPARγ activity was much greater than the GW9662 antagonist (Figure 7A). Moreover, when PPARγ was co-treated with BZ26 and GW 1929, the activation of PPARγ by GW1929 was inhibited by BZ26, suggesting that BZ26 competed with GW1929 for binding to the receptor (Figure 7B). To explore whether BZ26 could directly bind to PPARγ, we used the quantitative ITC method to measure the binding affinity and binding stoichiometry between PPARγ and BZ26 at 298 K. Fitting of the ITC data to a one-site binding model revealed three potential BZ26 binding sites within PPARγ with moderate binding affinity (*K*_a_ = 8.70 × 10^3^ ± 2.04 × 10^3^ M^–1^) (Figure 7C).

The structure of PPARγ comprises an A/B domain as well as C (DNA-binding domain), D (hinge), and F (ligand-binding domain, LBD) domains. To identify which amino acids of PPARγ are involved in binding BZ26, three different C-terminal deletion mutants (C/F, D/F, F) of PPARγ, the design and preparation of which are described in our previous report (Feng et al., 2016), were used. The data showed that BZ26 partially inhibited the activation of the C/F (107aa-475aa) mutant, suggesting that the BZ26 did not bind with the A/B mutant. In contrast, the D/F (181–475 aa) mutant could not be inhibited by BZ26, suggesting that amino acids 107aa-181aa in PPARγ were responsible for binding BZ26 (Figure 7D). However, BZ26 also partially inhibited the activation of the F (289–475 aa) mutant, suggesting that amino acids 289–475 aa were also required for their binding. Taken together, the 107aa-475aa maybe necessary for BZ26 binding with PPARγ.

## 4 Discussion

A large number of pre-clinical and clinical trials have shown that obesity is associated with the development of 13 different types of cancers, including breast cancer, prostate cancer, and ovarian cancer. Obesity also portends worse cancer-specific outcomes after diagnosis in several tumor types and significantly increasing human mortality (Renehan et al., 2006). However, the precise mechanisms linking obesity and cancer are not yet well-understood. Obesity is a pathological condition accompanied by excessive fat deposition in AT. Most cancers develop and metastasize in the vicinity of an AT-rich environment. Several reports have shown that adipocyte progenitor cells rapidly proliferated and differentiated into mature adipocytes in obese individuals, thereby contributing to tumor progression (Brown and McIntosh, 2003; Naugler and Karin, 2008). In this study, we found that mature adipocytes from the adipose tissue of HFD-fed mice promoted breast cancer progression, and co-injection of mature adipocytes and E0771 breast cancer cells into the HFD-fed mice further promoted obesity-related tumor progression. Based on these results and literature precedent, mature adipocytes in AT may be a crucial link between obesity and cancer (Nieman et al., 2011; Park et al., 2011).

CAAs are delipidated, cancer cell reprogramed-adipocytes that support tumor growth and survival by secreting excess inflammatory cytokines and proteases to create an environment that increases the invasiveness and aggression of cancer cells. Interestingly, in this study, we found that in the HFD-fed mice, mature adipocytes exhibited a CAA-like phenotype while the pre-adipocytes isolated from the visceral fat of HFD-fed mice did not emerge a CCA phenotype. In addition, in obesity, mature adipocytes are indicated to play a significant role in cancer cell proliferation, invasion, and metastasis (van Kruijsdijk et al., 2009; Park et al., 2011; Prieto-Hontoria et al., 2011; Biton et al., 2014). One study confirmed that CAAs were involved in breast cancer proliferation and metastasis (Wu et al., 2019). Here, we found that inhibition of PPARγ by BZ26 inhibited the differentiation of mature adipocytes to CAAs, thereby inhibiting obesity-related inflammation and breast cancer growth and metastasis. Therefore, inhibiting the differentiation of mature adipocytes to CAAs is a promising therapeutic strategy for treating obesity-related metabolic disorders and cancers by inhibiting the reprogramming of mature adipocytes to CAAs.

PPARγ is a ligand-dependent nuclear transcription factor that regulates adipocyte differentiation, and its dysfunction is believed to cause numerous life-threatening diseases such as diabetes and cancer. Reports have indicated that deletion of PPARγ in AT of obese mice protected the mice against HFD-induced obesity and insulin resistance (Jones et al., 2005; Feng et al., 2014; Landgraf et al., 2020). Moreover, perturbation of PPARγ signaling with novel PPARγ agonists and antagonists is gaining attention as a strategy for the treatment of several cancers, including breast cancer (Ahmed et al., 2007; Chandra et al., 2017). Yang et al. (2020) observed that inhibiting the activation of PPARγ prohibited the uptake of fatty acids by Nur77, which suppressed breast cancer progression. However, the role of PPARγ in the development of obesity-related cancer is still unclear.

In this study, we found the mature adipocytes in the visceral fat of HFD-fed mice have a CAAs phenotype but the stromal vascular fraction (SVF) of the visceral fat has not. Importantly, we found the derivate of the potent PPARγ antagonist GW9662, BZ26 inhibited the reprogramming of mature adipocytes in the visceral fat of HFD-fed mice into CAA-like cells and inhibited the proliferation and invasion of obesity-related breast cancer. Further study found that it mediated the browning of visceral, subcutaneous and perirenal fat and attenuated inflammation of adipose tissue and metabolic disorders. For the mechanism, we found that BZ26 bound and inhibited PPARγ by acting as a new modulator. Therefore, BZ26 serves as a novel modulator of PPARγ activity, that is, capable of inhibiting obesity-related breast cancer progression by inhibiting of CAA-like cell formation, suggesting that inhibiting the reprogramming of mature adipocytes into CAAs or CAA-like cells may be a potential therapeutic strategy for obesity-related cancer treatment.

## Data Availability

The data presented in the study are deposited in the Gene Expression Omnibus repository, accession number GSE240082.
