# Supplementary material for: Inhibition of PPARγ by BZ26, a GW9662 derivate, attenuated obesity-related breast cancer progression by inhibiting the reprogramming of mature adipocytes into to cancer associate adipocyte-like cells
Source: Front Pharmacol. 2023 Aug 15;14:1205030. doi: 10.3389/fphar.2023.1205030 (PMC10462981; doi:10.3389/fphar.2023.1205030)
Supplement: Supplementary file 1 [file Table1.DOCX]

| **Gene** | **Forward Primer** | **Reverse Primer** |
| --- | --- | --- |
| IL-1b | CTTCAGGCAGGCAGTATCACTC | TGCAGTTGTCTAATGGGAACGT |
| IL-6 | ACAACCACGGCCTTCCCTAC | TCTCATTTCCACGATTTCCCAG |
| Tnf-a | CGAGTGACAAGCCTGTAGCCC | GTCTTTGAGATCCATGCCGTTG |
| β-Actin | TGCTGTCCCTGTATGCCTCT | TTTGATGTCACGCACGATTT |
| Fizz1 | AGGAGCTGTCATTAGGGACATC | GGATGCCAACTTTGAATAGG |
| Ym1 | AGAAGGGAGTTTCAAACCTGGT | GTCTTGCTCATGTGTGTAAGTGA |
| Arg-1 | CTCCAAGCCAAAGTCCTTAGAG | AGGAGCTGTCATTAGGGACATC |
| CCR2 | ATGCAAGTTCAGCTGCCTGC | ATGCCGTGGATGAACTGAGG |
| NOS2 | GCTTCTGGTCGATGTCATGAG | TCCACCAGGAGATGTTGAAC |
| Adiponectin | GGAACTTGTGCAGGTTGGAT | GCTTCTCCAGGCTCTCCTT |
| Cidea | ATCACAACTGGCCTGGTTACG | TACTACCCGGTGTCCATTTCT |
| Cox8b | GAACCATGAAGCCAACGACT | GCGAAGTTCACAGTGGTTCC |
| Cox3 | CAAGGCCACCACACTCCTATT | GTCAGCAGCCTCCTAGATCA |
| Cox5b | TCTAGTCCCGTCCATCAGCA | AGACATTCTGTGAGGCAGGT |
| Pgc-1a | CCCTGCCATTGTTAAGACC | TGCTGCTGTTCCTGTTTTC |
| Ucp1 | CACCTTCCCGCTGGACACT | CCCTAGGACACCTTTATACCTAATGG |
| Fabp4 | GCTTTTGTAGGTACCTGGAAACTT | ACACTGATGATCATGTTAGGTTTGG |
| *Dio2* | TACAAACAGGTTAAACTGGGTGAAGATGCTC | GAGCCTCATCAATGTATACCAACAGGAAGTC |
| PPARα | AACATCGAGTGTCGAATATGTGG | CCGAATAGTTCGCCGAAAGAA |
| Otop1 | ACTCTCTGGTTGACAGTCGC | TGTGAGTCTCCACTTGCACC |
| Leptin | GTGGCTTTGGTCCTATCTGTC | CGTGTGTGAAATGTCATTGATCC |
| MMP-9 | GGACCCGAAGCGGACATTG | CGTCGTCGAAATGGGCATCT |
| MMP-11 | CCACTCACTTTCACTGAGGTG | CGTCAAACGGCAAGTTGTCAC |
| FABP2 | GTGGAAAGTAGACCGGAACGA | CCATCCTGTGTGATTGTCAGTT |
| PAI-1 | CAAGCTCTTCCAGACTATGGTG | ACCTTTGGTATGCCTTTCCAC |

**Table 1 sequences of primers used in this paper**
